# Rhode Island Lunatic Asylum

**Published:** 1845-02

**Authors:** 


*Rhode Island Lunatic Asylum.*—The site has been selected, and a valuable farm, consisting of from fifty to an hundred acres, has been purchased for the Asylum. It is situated about half way between the city of Providence and Pawtucket. The location is said to be a good one, combining variety and beauty of prospect, with retirement. We understand that suitable buildings will soon be erected.—*Ibid.*

